# Morphometric similarity deviations in stimulant use disorder point towards abnormal brain ageing

**DOI:** 10.1093/braincomms/fcac079

**Published:** 2022-03-28

**Authors:** Peter Zhukovsky, George Savulich, Sarah Morgan, Jeffrey W. Dalley, Guy B. Williams, Karen D. Ersche

**Affiliations:** Department of Psychology, University of Cambridge, Cambridge, UK; Department of Psychiatry, University of Cambridge, School of Clinical Medicine, Cambridge, UK; Department of Psychiatry, University of Cambridge, School of Clinical Medicine, Cambridge, UK; Department of Psychiatry, University of Cambridge, School of Clinical Medicine, Cambridge, UK; Department of Computer Science and Technology, University of Cambridge, Cambridge, UK; The Alan Turing Institute, London, UK; Department of Psychology, University of Cambridge, Cambridge, UK; Department of Psychiatry, University of Cambridge, School of Clinical Medicine, Cambridge, UK; Department of Clinical Neurosciences, University of Cambridge, Cambridge, UK; Wolfson Brain Imaging Centre, Cambridge Biomedical Campus, Cambridge, UK; Department of Psychiatry, University of Cambridge, School of Clinical Medicine, Cambridge, UK; Department of Systems Neuroscience, University Medical Centre Hamburg-Eppendorf, Hamburg, Germany

**Keywords:** morphometric similarity, cocaine, paired associates learning, orbitofrontal cortex, human connectome

## Abstract

Chronic drug use negatively impacts ageing, resulting in diminished health and quality of life. However, little is known about biomarkers of abnormal ageing in stimulant drug users. Using morphometric similarity network mapping, a novel approach to structural connectomics, we first mapped cross-sectional morphometric similarity trajectories of ageing in the publicly available Rockland Sample (20–80 years of age, *n* = 665). We then compared morphometric similarity and neuropsychological function between non-treatment-seeking, actively using patients with stimulant use disorder (*n* = 183, mean age: 35.6 years) and healthy control participants (*n* = 148, mean age: 36.0 years). The significantly altered mean regional morphometric similarity was found in 43 cortical regions including the inferior and orbital frontal gyri, pre/postcentral gyri and anterior temporal, superior parietal and occipital areas. Deviations from normative morphometric similarity trajectories in patients with stimulant use disorder suggested abnormal brain ageing. Furthermore, deficits in paired associates learning were consistent with neuropathology associated with both ageing and stimulant use disorder. Morphometric similarity mapping provides a promising biomarker for ageing in health and disease and may complement existing neuropsychological markers of age-related cognitive decline. Neuropathological ageing mechanisms in stimulant use disorder warrant further investigation to develop more age-appropriate treatments for older people addicted to stimulant drugs.

## Teaser

Using morphometric similarity, a novel measure of structural connectomics, Zhukovsky *et al.* report deviations in cortical regions in patients with stimulant use disorder consistent with accelerated brain ageing. Impaired associative learning was also shown. Brain ageing is a target for developing age-appropriate treatments for older people addicted to stimulant drugs.

## Graphical Abstract

**Graphical Abstract:**
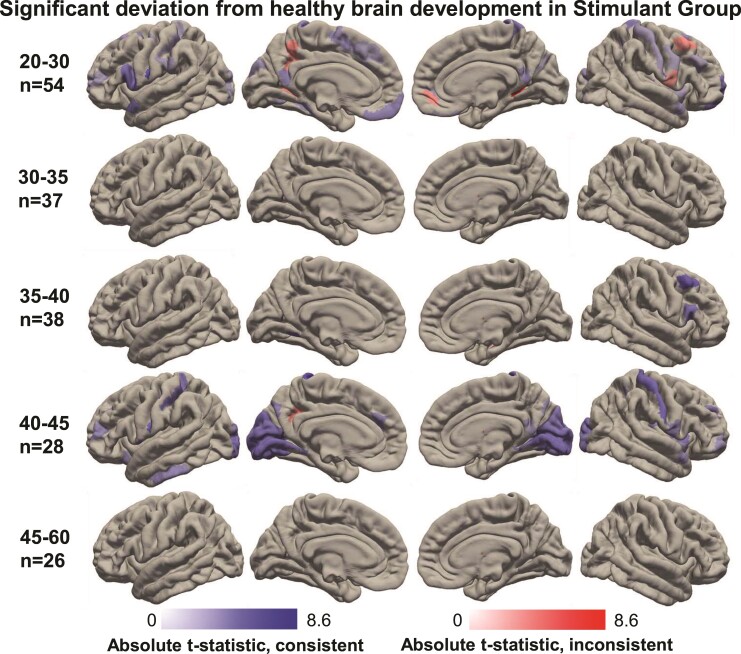
Abnormal ageing in stimulant use disorder.

## Introduction

Human ageing poses significant challenges to public health. As life expectancy in developed countries continues to rise,^1^ new measures in support of the quality of life and well-being of older people are urgently needed. In recent years, drug use by people aged 50 years and older has increased^2–4^ alongside a growing number of older people seeking specialist treatment for drug use.^5^ This pattern likely reflects ageing of the baby boomers (born between 1946 and 1964) who were exposed to more extreme drug use than any prior generation,^6^ whereas young drug users show impairments in executive functions that are typically associated with older age,^7–9^ cognitive deficits in older drug users are often mistaken for depression or the first symptoms of dementia.^10,11^

Structural brain changes in middle-aged stimulant drug users share a similar neuropathology with biological ageing.^12^ For example, age-related grey matter decline in prefrontal and temporal regions^13–15^ and cortical thinning in the angular gyrus and insula^16^ have been shown in individuals addicted to stimulant drugs such as cocaine or amphetamines. Frontotemporal atrophy has also been found in polysubstance users including stimulant drugs.^17–19^ Of particular interest, volume loss in cocaine-dependent individuals was at almost *twice* the annual rate compared with healthy controls.^14^ Together, these studies suggest that brain ageing processes may be accelerated in individuals with harmful regular stimulant drug use. Identifying biomarkers of abnormal brain ageing in older people addicted to stimulant drugs would thus help establish their needs as a more accurate reflection of biological rather than calendar age.

Brain network integrity and connectivity typically decreases with healthy ageing and deteriorates further in neurodegenerative diseases.^20^ However, little is known about the impact of chronic stimulant drug use on age-related brain network trajectories from young adulthood to older age. Morphometric similarity (MS) network mapping is a novel technique that allows for the construction of individual whole-brain networks using structural MRI.^21–23^ This method quantifies the similarity between two cortical regions in terms of multiple morphometric features (e.g. cortical thickness). Prior work in the macaque monkey has shown that regions which have high MS are also more likely to be connected by an anatomical tract using gold standard tracing methods.^21^ This technique has also been used to show reduced interconnectedness in frontal and temporal cortical areas in patients with psychosis, which was highly replicable across three data sets.^22^ MS mapping therefore provides an estimate of anatomical connectivity and can capture known cortical cytoarchitecture and patterns of gene expression across the brain.^21^

We aimed to investigate the extent to which stimulant drug use may alter brain-ageing processes in chronic users compared with healthy control participants using MS mapping. Our objectives were as follows: (i) to establish normal age-related trajectories in MS networks in a large, publicly available normative sample; (ii) to compare age-related changes in MS networks between stimulant use disorder (SUD) patients and healthy controls; (iii) to characterize deviations from normative MS trajectories in SUD patients; and (iv) to relate paired associates learning, a neuropsychological marker of normal ageing,^24^ with MS deviations in whole-brain analyses. We operationalize abnormal brain ageing by investigating MS deviations from normative values that resemble MS values of healthy older participants. Since we did not have longitudinal data, we refrain from direct conclusions about abnormal ageing. We predicted aberrant regional MS in SUD patients compared with normative trajectories, consistent with an accelerated profile of brain ageing. We further predicted that unlike healthy controls, patients with SUD would show a paired associates learning deficit consistent with the notion of accelerated ageing.

## Materials and methods

### Samples

#### Rockland Sample

Cross-sectional data from 665 individuals was obtained from the Nathan Kline Institute (Rockland Sample, NKI-RS, http://fcon_1000.projects.nitrc.org/indi/enhanced/index.html) to construct age-related trajectories for MS measures. The Rockland Sample is an ongoing initiative for curating a large, openly available data set across the lifespan.^25^ T_1_ MRI data registration in *Freesurfer* (v5.3.0) was quality controlled by visual inspection.

#### Cambridge sample

This study included previously acquired data sets, comprising 395 T_1_-weighted MR images from four cross-sectional studies conducted between 2006 and 2014.^26–31^ Ethical approval was obtained from National Research Ethics Committees and consent included the re-use of data for further analyses in all studies. All participants underwent MR brain scans at the Wolfson Brain Imaging Centre, University of Cambridge, UK, using a Siemens TIM Trio 3T system (see Supplementary material).

The total sample (*n* = 331) comprised 148 healthy control participants and 183 stimulant-addicted patients. All participants with a history of chronic stimulant drug use satisfied the Diagnostic and Statistical Manual 4th Edition Text Revision (DSM-IV-TR) criteria^32^ for dependence on either cocaine (89%) or amphetamines (11%). Stimulant drug users were actively using, as verified by urine screen, with an average of 14.5 years (± 7.7 SD) of use, starting at the age of 19 years (± 5.4 SD). The majority of the SUD group also met the DSM-IV-TR criteria for dependence on another substance (37% opiates, 23% alcohol and 9% cannabis). SUD diagnosis was ascertained using the Structured Clinical Interview for DSM-IV. The 95% of the SUD group that were tobacco smokers had been for an average of 21.2 years ( ± 8.9 SD), with a mean starting age of 13 years ( ± 3.6 SD). Participants with a history of psychotic disorders or traumatic brain injury were excluded. Control participants were in good health and had never met DSM-IV-TR criteria for substance dependence. For further demographic details, see Supplementary material, Section 1.

### Procedures

#### Morphometric similarity network mapping and behavioural performance

Construction of MS networks followed previous studies,^21^ with more details available in the Supplementary material. Briefly, MS networks were built using *Freesurfer*-derived features including (i) grey matter volume, (ii) surface area, (iii) average cortical thickness, (iv) mean curvature, (v) Gaussian curvature, (vi) folding index and (vii) intrinsic curvature index. For each participant, each feature was normalized (mean centred with a standard deviation of 1) and Pearson’s correlations were computed between features derived for each of the 360 regions. Columns of the resulting 360 × 360 MS matrix were then averaged to obtain 360 mean regional MS values. Regional MS provides a measure of that region’s mean similarity to all other brain regions and is equivalent to the weighted degree or hubness of that region.^21, 22^ Paired associate learning (PAL) was assessed in the Cambridge sample using the Cambridge Neuropsychological Test Automated Battery Paired Associates Learning test (https://www.cambridgecognition.com/cantab/cognitive-tests/memory/paired-associates-learning-pal/).^33^ Participants first observed different colourful geometric patterns randomly appearing in white boxes on a 2D touch-screen computer. Subsequently participants were asked to remember the locations of these patterns by pressing the box in which they previously appeared, meaning participants had to deliberately encode the association between several patterns and their location. Participants were reminded of their locations if an error was made. The level of difficulty of the trial increases throughout the task. The main outcome measures were first trial memory score (the number of patterns correctly located on the first trial in each stage)^33^ and the total number of errors made across all completed trials, an index of memory decline.^24^

#### Normal ageing from young adulthood to later life (Rockland Sample)

Mean regional MS in the Rockland Sample was used to model normal ageing and assess deviations in each region of interest in the SUD group. Linear, quadratic and cubic mappings from age to regional MS were considered (see Supplementary material, Section 5 for trajectory fitting).

Four separate categories were formed based on the trajectory slope (i.e. the first derivative of the quadratic trajectory) in early (age = 27 years) and later life (age = 60 years): increasing, i.e. increasing at both ages; decreasing, i.e. decreasing at both ages; convex, i.e. increasing at 27 years and decreasing at 60 years; and concave, i.e. decreasing at 27 years and increasing at 60 years. This categorization was used to assess whether deviation from the normative trajectory was consistent with an accelerated ageing profile (details below). Trajectories for 48 regions had negative adjusted r^2^ values and were excluded as the fitted functions failed to explain a sufficient amount of variance in regional mean MS (Supplementary material, Section 5).

### Statistical analyses

#### Abnormal morphometric similarities associated with stimulant use disorder

Mean regional MS was compared between the healthy control and SUD groups (both from the Cambridge sample) using parametric independent samples *t*-tests. Both groups were matched for age (Supplementary material, Table 1). Consistent with European drug use surveys,^34^ our sample was predominantly male but balanced between groups. Hence, these variables were not included as covariates. We also computed group differences in regional MS measurements in the same way. Two-tailed *p*-values from 360 *t*-tests (testing each region individually) were corrected for multiple comparisons using false discovery rate (FDR), *P* < 0.01 (*mafdr* function in MATLAB R2016b).

#### Deviations from normal brain ageing (Cambridge sample)

For each of the healthy and SUD participants, deviations from age-related trajectories were calculated by subtracting their regional mean MS from the regional mean MS predicted by the trajectory for their age (Supplementary material, Section 5). Participants’ MS deviations were grouped into five age categories: 20–30, 30–35, 35–40, 40–45 and 45–60 years of age in order to achieve a balance between equal length of each age category and the number of participants included in each category. One sample *t*-tests were used to determine whether MS deviation in each age category was significantly different from zero. In each age category, the *P*-values from 360 *t*-tests (testing each region individually) were corrected for multiple comparisons using FDR, *P* < 0.01 and permutation testing (Supplementary material, Section 4).

**Figure 1:**
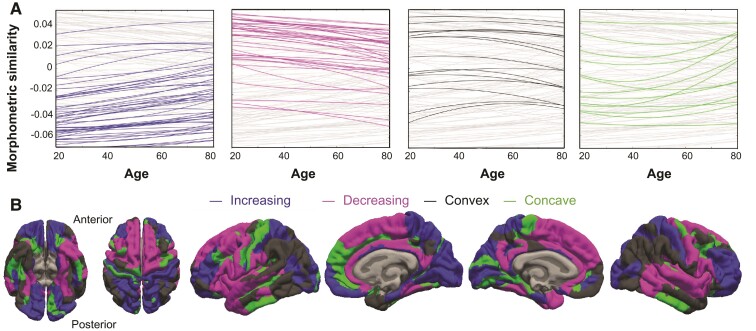
**Cross-sectional trajectories of MS in a normative sample (NKI-RS, Nathan Kline Institute, Rockland Sample).** Increasing (blue), decreasing (red), convex (black) and concave (green) trajectories were observed (**A**), with regions showing particularly high and low mean similarity scores tending towards zero with increasing age. All trajectories are plotted in the background in grey. The anatomical distribution of increasing, decreasing, convex and concave trajectories is shown in (**B**).

MS deviations from decreasing trajectories were deemed consistent with accelerated ageing if these deviations were less than zero. Deviations from increasing trajectories were consistent with accelerated ageing if these deviations were greater than zero. If the trajectory was convex and its turning point corresponded to an age that exceeds the maximum age in a given category, positive deviations from this trajectory were consistent with accelerated ageing. Conversely, if the turning point of a convex trajectory corresponded to an age that is below the minimum age in a given category, negative deviations from this trajectory were consistent with accelerated ageing. If the trajectory was concave, then the location of the tipping point of this trajectory would determine whether positive or negative deviations were consistent with accelerated ageing (Fig. 1; Supplementary material, Sections 5 and 6). For instance, in young participants (20–30 years), positive deviations from convex trajectories (increasing in early life) and negative deviations from concave trajectories (decreasing in early life) were consistent with accelerated ageing.

To validate our region-wise ageing analysis, we used more traditional measures of brain age gaps.^35,36^ We trained a lasso regression model to estimate age from the mean MS in the Rockland data set and then applied this model to SUD and healthy control participants to estimate their ‘brain age’ (Supplemental material, Section 8). Brain age gaps were calculated by subtracting predicted ‘brain’ age from calendar age.

#### Associations between brain and neuropsychological markers of ageing (Cambridge sample)

To test the relationship of mean MS with age and PAL performance, we used multivariate partial least squares (PLSs) regression (MATLAB R2016b). Age was included as an outcome variable to separate the variance in MS that was related to age versus the variance related to PAL performance across all participants. PAL total errors were included as an indicator of pathological ageing and learning impairment, respectively. Data from 115 healthy control participants and 115 SUD patients were available. In the (230 × 360) predictor matrix in the first PLS regression, each row represented a participant and each column a region of interest, with each matrix entry being the mean MS for a given participant and a region of interest. The outcome was a 230 × 2 matrix with the two columns given by age and PAL total errors. Permutation testing was used to determine whether the PLS component scores explained a significant amount of variance in the outcome matrix, whereby 5000 randomized PLS regressions were ran. Permutation testing further allowed us to test if the significant PLS component scores were associated with age and PAL total errors, a measure of learning and memory.

### Data availability

The MS derivatives and demographic data is available at https://www.repository.cam.ac.uk/handle/1810/333884

## Results

### Participant characteristics

As shown in Supplementary material, Table 1, participants were predominantly male in the Cambridge groups [90% and 81% male in SUD and control groups, respectively; *χ*²=4.89, *P* = 0.03] and aged between 18 and 59 years [mean age 36 years in both groups; *t*(329) = 0.45, *P* = 0.65]. The healthy control group had significantly higher estimated verbal intelligence compared with the SUD group [*t*(314) = 6.36, *P* < 0.001]. As impulsivity is a hallmark of addiction,^32,37,38^ the SUD group showed significantly higher impulsivity total scores on the BIS-11 compared with the control group [*t*(315) = −16.09, *P* < 0.001]. Participants in the Rockland Sample were predominantly female (66%) and had a mean age of 37.4 years, which did not differ significantly from the Cambridge healthy control group [*t*(644) = −1.17, *P* = 0.24].

**Figure 2:**
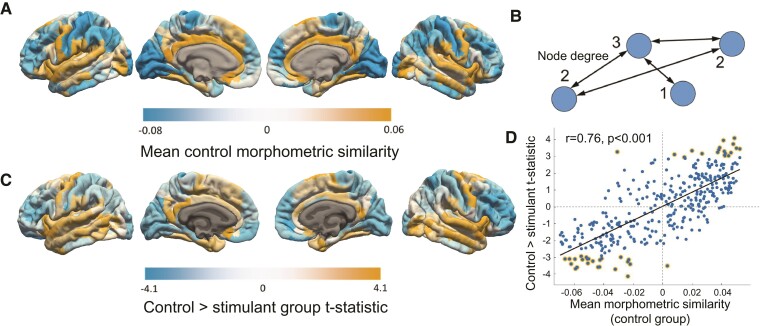
**Case–control differences in SUD**. Mean MS in healthy control participants (**A**) was significantly different from the mean MS in patients with SUD in 43 regions (**C**) (Supplementary material, Table 3). Orange colours indicate greater MS in controls and blue colours indicate greater MS in SUD (**C**). Significant differences between the control and SUD groups were observed in those regions with particularly high or low MS scores (**D**, golden circles show significant differences); the SUD group tended to have MS scores closer to zero (**D**, *P*_PERMUTATION_ = 0.002). Mean MS can be conceptualized mathematically as weighted regional degree as illustrated in (**B**).

The cortical map of regional MS (Fig. 2A; Supplementary material, Fig. 1) averaged across the Rockland Sample was highly correlated with the cortical map of regional MS averaged across the Cambridge control group (Pearson’s correlation coefficient *r* = 0.98, *P* < 0.001). This map also strongly resembles healthy cortical MS maps from prior studies^21,22^ (Supplementary material, Section 3).

#### Age-related brain changes in healthy controls

For each cortical region, we modelled mean MS in the Rockland Sample as a function of age. Quadratic and cubic models significantly improved model fit compared to linear models (see Supplementary material, Section 5). Quadratic age-related trajectories were selected to account for nonlinear associations between age and mean regional MS based on model fit analyses. Cubic trajectories did not improve the model fit with respect to quadratic trajectories and were not as easily interpretable.

Age-related trajectories for all cortical regions are plotted in Fig. 1A, grouped into four categories: increasing (121 regions), decreasing (119 regions), convex (57 regions) and concave (63 regions). Fig. 1B shows the cortical locations of regions that followed these four different trajectories.

#### Age-related brain changes in stimulant use disorder

We observed significant case–control differences in 43 regions after FDR < 0.01 multiple comparison correction (Fig. 2C; Supplementary material, Table 4) and in 60 regions after permutation testing in which group labels were randomly reassigned (Supplementary material, Fig. 9). Regions with significantly reduced MS in the SUD group included the inferior frontal gyrus (IFG), insular cortex, orbital frontal cortex (OFC), middle frontal gyrus (MFG), pre- and postcentral gyri, anterior temporal, superior parietal and occipital areas.

There was a significant correlation between the cortical map of case-control MS differences and the cortical map of regional MS in the Cambridge healthy control participants (*r* = 0.76, *P* < 0.001, Fig. 2A and D). Specifically, regions with high MS in controls (network ‘hubs’, Fig. 2B) were most likely to show reduced MS in SUD cases, whilst regions with low MS in control participants were most likely to show increased MS in SUD patients.

**Figure 3:**
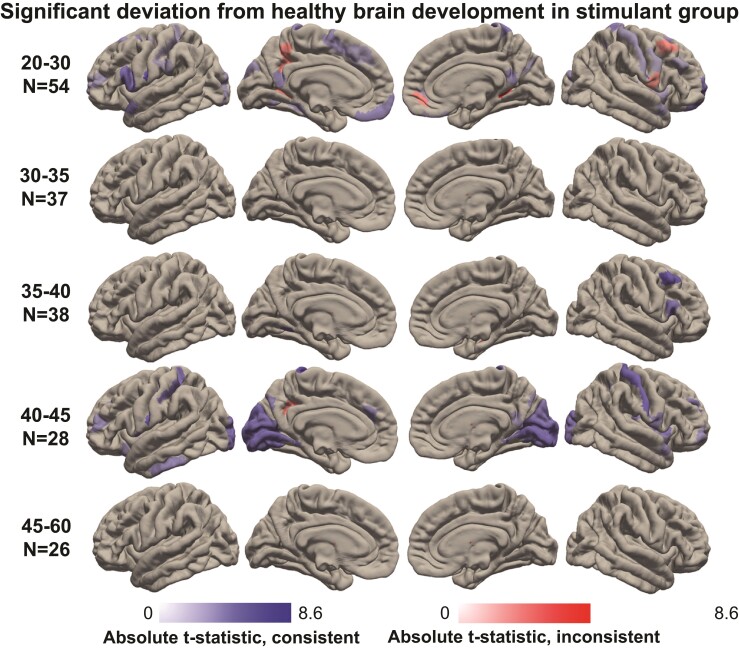
**Significant deviation from healthy brain development in SUD.** Significant mean MS deviations from normative development in five age categories in patients with SUD. Significance was determined using one-sample *t*-tests, FDR corrected at *P* < 0.01. Five age categories comprised 20–30-, 30–35-, 35–40-, 40–45- and 45–60-year-old participants. Those regions for which the quadratic trajectory provided a poor fit to the data (adjusted *r*^2^<0, see Supplementary material, Fig. 6) were excluded. Deviations (absolute *t*-statistics) consistent with accelerated ageing are shown in blue, whereas those inconsistent with ageing are shown in red (see Supplementary material, Fig. 7 for the deviations in the Cambridge control group).

We investigated abnormal ageing associated with SUD by comparing the Cambridge SUD patients to the normative MS trajectories derived from the healthy Rockland Sample. Figure 3 (and Supplementary material, Table 5) shows the cortical regions that showed deviations in patients with SUD, grouped into five age categories. Across all age categories, we observed 104 cortical regions that exhibited significant deviations. Importantly, 88% of these regions showed deviations that were consistent with an accelerated ageing profile.

The most widespread deviations from healthy brain ageing were found in the youngest SUD patients (20–30 years; 57 regions), as well as in the older SUD patients (40–45 years; 38 regions). More localized, yet robust MS deviations were also found in the 35–40-year-old SUD patients (8 regions). Significant deviations in the MFG, IFG and insular subregions were observed in younger (20–30 years) as well as older (35–40 and 40–45 years) SUD patients. On the other hand, significant differences in the occipital cortices were specific to the 20–30- and 40–45-year-old SUD patients. The frontal pole, pre- and postcentral gyri also showed significant deviations from healthy ageing.

Notable departures from an abnormal ageing profile were found in a subregion of the precuneus and the posterior cingulate of young SUD patients. These regions showed significant age-related deviations, but in the opposite direction to that expected from accelerated ageing.

Finally, when comparing the Cambridge control group to normative age-related trajectories in the Rockland Sample, only 22 regions exhibited significant deviations (compared with the 104 significant deviations exhibited in the Cambridge SUD group). Hence, we expect that the majority of deviations from normative MS trajectories observed in our SUD patients to be related to SUD and not attributable to methodological differences between the Cambridge and Rockland data sets (e.g. different MR acquisition protocols).

#### Brain age gaps

Consistent with an abnormal ageing profile in the SUD group found above, we also report brain age gaps that were significantly higher in SUD than in the control group (+3.3 years; Supplementary material, Section 8).

#### Association between MS and paired associates learning performance

We found main effects of group [control versus SUD, *F*(1,227) = 85.2, *P* < 0.001] and age [*F*(1,227) = 20.0, *P*= < 0.001] on the PAL test, as patients with SUD and older participants made significantly more errors when trying to remember the spatial locations of geometric patterns. No group-by-age interactions for PAL outcome measures were observed (Supplementary material, Table 2). Although all age groups showed significant between-group differences in total errors (all *P*’s < 0.03), the effect size in the participants 40–60 years of age was the smallest (*d* = 0.7). Effect sizes (Cohen’s *d*) for the control versus SUD difference in each age category were *d*_20–30_ = 1.6; *d*_30–35_ = 1.1; *d*_35–40_ = 1.2; *d*_40–45_ = 1.6; *d*_45–60_ = 0.7.

**Figure 4:**
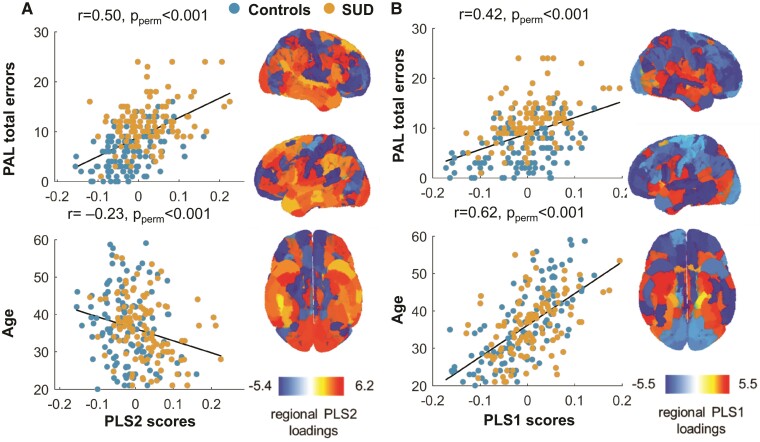
**Brain-cognition association between MS and paired associates learning**. Relationship between PAL, age, MS and stimulant drug use. The first two components of the PLS regression jointly explained 43.4% of variance in PAL total errors and age, and 4.35% of variance in regional MS in all 360 regions of interest. PLS2 explained a significant amount of variance in PAL performance and age (15.5%, *P*_perm_ = 0.02) but showed a strong positive association with PAL errors and a weak negative association with age (**A**). PLS1 also explained a significant amount of variance in PAL performance and age (27.9%, *P*_perm_ = 0.02) and was positively associated with both of these variables (**B**). The pattern of regional MS loadings on PLS1 (*r* = 0.385, *P*_perm_ = 0.37) and PLS2 (*r* = 0.165, *P*_perm_ = 0.44) was distinct from the pattern of the control versus stimulant use disorder group difference *t*-statistics (see Fig. 1C).

The first two PLS components jointly explained 43.4% of the variance in learning and age. PLS1 accounted for 27.9% of the variance and was positively associated with both age (*r* = 0.62, *p* < 0.001) and PAL total errors (*r* = 0.42, *P* < 0.001, Fig. 4B). PLS2 accounted for 15.5% of the variance and showed a strong positive association with total errors (*r* = 0.50, *P* < 0.001) and a weak negative association with age (*r* = −0.23, *P* < 0.001) (Fig. 4A). PLS component maps were not significantly correlated with the SUD-control *t*-statistic difference maps after permutation testing.

PLS1 regional loadings showed that reduced MS in large portions of the prefrontal and parietal cortices were associated with greater PLS1 scores and thus older age and more errors on the PAL test. PLS2 regional loadings showed that increased MS in large portions of temporal, parietal lobes and dorsal prefrontal cortex was associated with greater PLS2 scores and thus more errors on the PAL test. Unlike PLS1 (*t* = 1.68, *P*_perm_ = 0.15), PLS2 component scores were significantly higher in the SUD group than in the control group (*t* = 5.4, *P*_perm_ < 0.001), thereby linking group differences in PAL performance and the association between regional MS and PAL performance. There were no significant differences between PLS1 component scores for control and SUD participants (*P*_perm_ > 0.05).

## Discussion

Older adults seeking treatment for drug-related problems are among the most vulnerable and fastest growing groups in society.^39^ Chronic stimulant drug use may accelerate ageing processes, resulting in diminished health and quality of life but little is known about the needs of these older drug users. Using MS mapping, we showed reduced MS in patients with SUD in-line with abnormal brain ageing in diverse cortical regions associated with both ageing and key phenotypes of SUD, including decreased similarity of frontal gyral regions.^40–42^ Moreover, patients with SUD showed a deficit in paired associates learning, a key neuropsychological marker of cognitive decline^24^ in healthy ageing, dementia and SUD. Deficits in the auditory-verbal memory domain have been previously observed in polysubstance use disorder.^43^

### Abnormal morphometric similarity in stimulant use disorder

We first established age-related trajectories from young adulthood (20–30 years) to mid-to-later life (45–60 years) for cortical regions using openly available normative data. Mean regional MS in the Rockland Sample converged towards zero with increasing age. Decline in cognitive performance in older adults, including reduced working memory or response inhibition has been attributed to atrophy of the prefrontal cortex,^44,45^ one of the key regions affected by healthy ageing.^46^ The patterns of change in regional MS observed in the Rockland Sample were consistent with previous studies showing reduced functional connectivity^47,48^ and age-associated white matter changes^49^ in similar anatomical networks, thus providing convergent validity for the normative trajectories that were used as a benchmark of normal ageing.

Previous studies have shown structural alterations in patients with SUD.^15,16,50^ We found reduced similarity in the Cambridge SUD group compared with the healthy control group in several brain regions including the OFC, IFG and insular cortices, in addition to anterior temporal, superior parietal and occipital areas. Reduced similarity in these areas is consistent with prior evidence showing decreased volume and cortical thickness in the OFC, insula and temporal cortices in SUD^52,30,51^ and decreased OFC volume found in individuals with polysubstance use disorder.^18^ More generally, reduced similarity in our SUD group compared with healthy control groups reflects previously reported evidence for highly similar regions showing reduced frontal and structural connectivity in neurological disorders,^53^ psychosis^22^ and pathological ageing.^54^ An alternative measure of structural connectivity that uses grey matter volume rather than a combination of measures of morphology used in MS is the profile similarity index. Using this measure, reduced connectivity of dorsolateral prefrontal regions has been shown in alcohol use disorder.^55^

### Profile of abnormal brain ageing

Previous studies have reported associations between prolonged stimulant drug use and grey matter decline in the IFG, medial and lateral OFC, cingulate and insular cortices.^15,30,56,57^ These studies raise the possibility that longer duration of stimulant drug use may interact with some of the brain regions affected by ageing. Our results are consistent with previous studies of ‘brain-age gaps’ suggesting that psychiatric conditions interact with the ageing process^35,36,58,59^ and provide a way of mapping deviations in MS to age-related cross-sectional trajectories. The MS patterns observed in our sample indicated large-scale structural connectivity changes in SUD consistent with abnormal brain ageing, specifically in the IFG, insula, MFG and occipital cortex. It is possible that chronic stimulant drug use may accelerate brain networks with overlapping susceptibility to the functional, structural and/or cognitive effects of ageing. For example, impairments in inhibitory control are associated with decreased activation in the IFG in drug users.^60^ Likewise older adults show less activation in this region during tasks of cognitive control compared with younger adults.^40^ Critically, our SUD group showed several significant deviations from normal ageing, whereas our healthy control group showed only very few significant deviations, suggesting that abnormal brain ageing was specific to SUD. We found that 20–30 year old SUD patients showed the most extensive pattern of MS deviations, but also observed similar alterations in middle-aged adults (35–40 and 40–45 years). Widespread age-related differences in MS point towards an abnormal and potentially pathological ageing profile; a notion supported by several lines of evidence. For example, molecular biomarkers of neurodegenerative diseases such as hyperphosphorylated tau^61,62^ and amyloid protein deposition^63^ have been found in young drug users. Evidence from peripheral biomarkers obtained from drug-addicted individuals have also shown altered immune and chronic hepatic inflammation processes consistent with abnormal ageing.^64^

### Morphometric similarity and paired associates learning

Paired associates learning decline exceeding impairments typically seen in healthy adults reaching older age (45–60 years) were found in young SUD patients, with middle-aged SUD patients (40–45 years) showing more severe impairment. While the performance of 45–60 year old patients was worse than that of control participants in the same age group, the case-control effect size was much smaller than in the younger age groups, indicating normal age-related decline in that PAL test. Impaired PAL performance is highly characteristic of dementia,^24^ but also prevalent in drug addiction.^7,65–67^ Difficulty remembering the spatial location of a given stimulus (i.e. associative learning) is related to structural changes in the hippocampal formation in the temporal lobe,^68^ a key area that is altered by the course of addiction^69^ and also among the first to be damaged in Alzheimer’s disease.^70^

### Implications and conclusions

Our study has important methodological strengths. MS network mapping is a novel yet robust technique useful for identifying abnormal brain networks relevant to neuropsychiatric disorders.^21–23^ We not only extended use of this method to SUD but also showed comparability of age-related changes in MS between two independent healthy control groups. Importantly, these networks were derived from T_1_ images using the same scanning protocol, therefore allowing re-examination of existing data. The cortical map of MS in our healthy control group was also highly associated with those reported in previous studies,^21,22^ confirming its replication in healthy individuals. Comparison of normative and patient data (i.e. using the Rockland Sample initiative) further allowed us to map MS trajectories of brain structure at different stages of adulthood as a means to investigate pathological biomarkers of abnormal ageing within an open science framework.

Our study also has important clinical implications. Firstly, it suggests that neurochemical biomarkers such as amyloid, tau and inflammatory markers already targeted in dementia and movement disorders^71^ may be of relevance to the treatment of SUD. Faster detection of biological age outpacing calendar age could also help prevent early onset diseases and premature mortality in older drug users.^12^ Finally, use of MS mapping as a biomarker of ageing alongside reliable and well-validated neuropsychological measures of cognitive decline could better differentiate the cognitive symptoms of SUD from those of dementia and depression.

The main limitation of our study is the lack of longitudinal imaging data. Whilst our results are in-keeping with accelerated brain ageing, longitudinal data are warranted to verify whether the abnormal trajectories seen in SUD patients is accelerated. Furthermore, the restricted number of neuropsychological measures limits full characterization of SUD-related cognitive impairment and their potential associations with abnormal ageing. Future research may want to expand on this using diffusion tensor imaging and resting-state MRI to investigate the relationships between abnormal MS, cognitive function, white matter and functional connectivity. Further, it would be important to clarify whether MS deviations indicative of abnormal ageing that we report here in active SUD patients persist following drug abstinence, as some evidence suggests that SUD-related cognitive^72,73^ and neuroanatomical^15,74^ impairments may recover. Impaired structural connectivity,^55^ neurocognition and response inhibition^43^ in individuals with alcohol use disorder may be restored after prolonged abstinence, although grey matter loss and dysconnectivity has been shown to persist in those individuals who relapse to drinking.^55^ Other studies, however, report frontotemporal atrophy in participants with alcohol and cocaine use disorder following 1–3 years of abstinence.^18^ Findings from analogous experimental approaches in animals would also be necessary to better understand the exact neuropathological processes by which stimulant drugs accelerate brain ageing.

In sum, we report reduced MS in-keeping with abnormal brain ageing in frontal and parietal cortical regions in patients with SUD. Our findings are of public health concern as they show that young people using stimulant drugs are potentially ageing faster than healthy adults. As drug addiction research generally focuses on early intervention and harm prevention strategies in adolescents and young adults,^75^ there is an unmet need to identify biomarkers of abnormal ageing in chronic drug users.^12,76^ As the number of older drug users is growing, we know little about their needs, which may be undetected and neglected. Our findings highlight that the needs of older drug users are likely greater than currently recognized because they may be exacerbated by accelerated ageing.

## Supplementary Material

fcac079_Supplementary_Data

## Abbreviations

DSM-IV-TR =: Diagnostic and Statistical Manual 4th Edition Text Revision
FDR =: false discovery rate
IF =: insular cortex; MS = morphometric similarity
MFG =: middle frontal gyrus
NKI-RS =: Nathan Kline Institute Rockland Sample
OFC =: orbital frontal cortex
PAL =: paired associate learning
PLSs =: partial least squares
SUD =: stimulant use disorder
TR =: repetition time
