# Supplementary material for: Morphometric similarity deviations in stimulant use disorder point towards abnormal brain ageing
Source: Brain Commun. 2022 Mar 28;4(3):fcac079. doi: 10.1093/braincomms/fcac079 (PMC9178962; doi:10.1093/braincomms/fcac079)
Supplement: fcac079_Supplementary_Data [file fcac079_supplementary_data.docx]

Supplementary materials

1. Datasets

Five datasets were used in this study, four of which were acquired at the Department of Psychiatry and an additional lifespan brain development dataset was acquired at the Nathan Kline Institute Rockland Sample (NKI-RS, <http://fcon_1000.projects.nitrc.org/indi/enhanced/index.html>). Data collected in Cambridge contain T1-weighted MPRAGE images from a TIM Trio Siemens 3T scanner ^1–3^.More detail on the demographics can be found in Supplementary Table 1.

1.1 Cambridge Datasets

Cambridge data sets comprised 395 T1-weighted magnetic resonance images from four ethically approved study protocols conducted between 2006–2014. Ethical approval was obtained from the National Research Ethics Committees and consent included for the re-use of the data for further analyses. Healthy controls were recruited by advertisement; stimulant drug users were recruited by referral, by advertisement and by word-of-mouth. All participants underwent MR brain scans at the Wolfson Brain Imaging Centre, University of Cambridge, UK, using a Siemens TIM Trio 3T system. T1-weighted MR scans were acquired using a magnetization-prepared rapid acquisition gradient-echo (MPRAGE) sequence (176 slices of 1 mm thickness, TR = 2300 ms, TE = 2.98 ms, TI = 900 ms, flip angle = 9°, FOV= 240 x 256). The MPRAGE data acquisition was consistent for the period that the scans were acquired. All MR images were screened for abnormalities by a specialist in neuroradiology. The MPRAGE data acquisition was consistent for the period that the scans were acquired. All MR images were screened for abnormal radiological appearance by a specialist in neuroradiology.

DTI data were not available on all participants, hence only T1 images were used for morphometric similarity (MS) analyses. All participants were screened for presence of psychiatric disorders using the Mini-International Neuropsychiatric Interview^4^. Participants with stimulant use disorder (SUD) showed higher trait impulsivity assessed using the Barratt Impulsivity Scale (BIS-11).

Age distributions were not significantly different (Supplementary Table 1). Further, age distribution in the SUD group did not differ significantly from a normal distribution (Kolmogorov-Smirnov distance=0.05, p>0.1), while in the control group the age distribution did show a significant deviation from normality (Kolmogorov-Smirnov distance=0.08, p=0.03). Age was strongly correlated with years of stimulant use (r=0.753, p<0.001, n=166, since years of use information was missing for some of the stimulant users). While this data was collected as part of several studies (“Study A”, “Study B”, “Study C” and “Study D”), the assessment methods and scanning parameters were similar across all participants.

The proportions of men and women in control and SUD groups were significantly different (X^2^=4.89, p=0.027). Cocaine use is more commonly found in men ^5^, hence our samples are predominantly male. Further, premorbid verbal intelligence measured using the National Adult Reading Test (NART) was significantly worse in stimulant users (t_141,174_=6.35, p<0.001). The SUD group also displayed higher trait impulsivity levels (t_141,174_=16.09, p<0.001; NART and BIS-11 scores were not available for some of the participants (15 missing NART scores and 14 missing BIS-11 scores).

1.2 Rockland Sample

Data from the Rockland Sample was acquired for 941 individuals in the age range of 6-85 years through a community-ascertained epidemiologic design ^6^, all of whom resided in Rockland County, USA (NY). Institutional Review Board Approval was obtained for at the Nathan Kline Institute (Phase I #226781 and Phase II #239708) and at Montclair State University (Phase I #000983A and Phase II #000983B). Since the purpose of this sample was to investigate cross-sectional developmental trajectories in 20-80-year olds, only those participants falling into this age category were included (n=665). MRI images were acquired on a 3T SIEMENS TIM Trio scanner, with a repetition time TR = 1900ms and an isotropic voxel size = 1mm (<http://fcon_1000.projects.nitrc.org/indi/enhanced/NKI_MPRAGE.pdf>). Limited demographic information (age, sex, and handedness) was publicly available in order to protect the anonymity of the participants. However, this information was deemed sufficient for constructing cross-sectional developmental trajectories in general population. High consistency between the Rockland data and the Cambridge control group supported the choice of sample for assessment of normative brain MS development (Supplementary Figure S3). Visual quality controls were performed to exclude T1 images with artefacts such as excessive amount of blurring. *Freesurfer* (version 5.3.0) segmentations were checked for segmentation accuracy.

1. MS network construction: methods and discussion

Briefly, T1-weighted MRI images were pre-processed using *Freesurfer* (recon-all). *Freesurfer* segmentations were checked for segmentation accuracy; any participants that did not pass visual quality control inspection of the T1w data were excluded. Next, 360 regions of interest from the cortical parcellation (Human Connectome Project, (Glasser *et al*, 2016)) were registered from the *fsaverage* (MNI305) space to individual subject space (*mri_label2label*). Next, estimates of 1) gray matter volume, 2) surface area, 3) average cortical thickness, 4) mean curvature, 5) Gaussian curvature, 6) folding index and 7) intrinsic curvature index were calculated using *mris_anatomical_stats* for each label. These seven morphometric features were z-scored to normalize different scales of these variables. The resulting 7x1 vectors were correlated using Pearson’s direct correlation coefficient for each pair of the 360 regions, resulting in a 360 x 360 pairwise correlation matrix. Analyses were based on T1 weighted images only, which may result in less precise estimates compared to analyses considering additional T2 and or DTI data ^8,9^. This matrix was demeaned to allow for better inter-individual comparisons. This procedure was repeated for participant data from the Rockland sample (MATLAB R2016b code is available on <https://github.com/peterzhukovsky/brain_ageing>). Seidlitz et al ^8^ showed that even 5 features derived from T1 weighted images are sufficient to construct MSN; here we used 7 features. In addition, our mean MSN look very similar to previously published MSN results, further supporting our MSN construction method ^10^.

Morphometric similarity network (MSN) mapping is a novel yet robust technique useful for identifying abnormal brain networks relevant to neuropsychiatric disorders ^8,10,11^. We not only extended use of this method to stimulant use disorder, but also showed comparability of age-related changes in MSNs between two independent healthy control groups. Importantly, these networks were derived from T1 images using the same scanning protocol, therefore allowing re-examination of existing data. It is worth noting that the cortical map of MS in our control group was highly similar to those reported in previous studies ^8,10^, confirming its replication in healthy individuals. Comparison of normative and patient data (i.e. using the Rockland sample initiative) also allowed us to map MSN trajectories of brain structure at different stages of adulthood as a means to investigate pathological biomarkers of abnormal ageing within an open science framework.

1. Reliability of MSN construction

MS analyses followed previous studies ^8,10^. Mean MS maps showed a great degree of similarity between the Cambridge control group, the Rockland sample and previously reported mean MS in healthy participants ^10^ (Supplementary Figure S1).


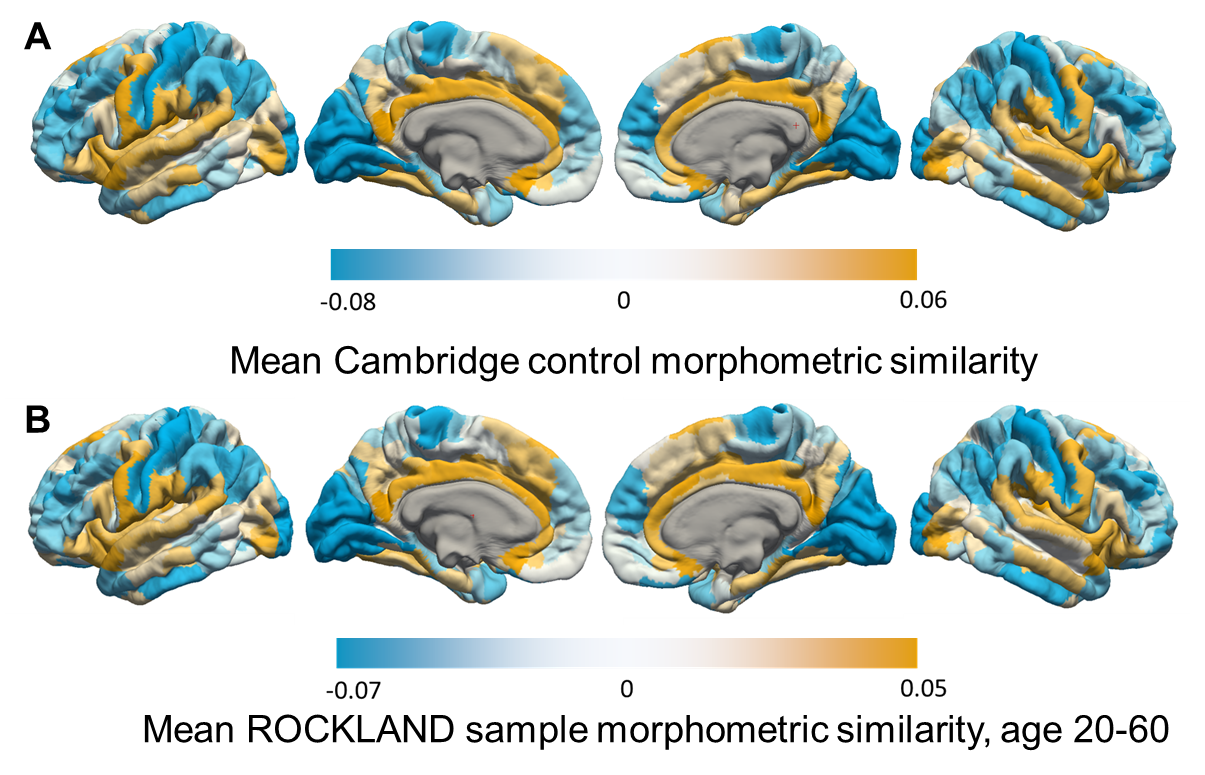
Supplementary Figure S1: A high degree of consistency was observed between mean MS in healthy controls in previous samples (i.e. Morgan et al 2019), the Cambridge control group (A) and publicly available Rockland dataset (B). Notably, the MS networks used in (Morgan et al 2019) were constructed with 10 different morphometric features, including FA, MD and MT measures in addition to those metrics derived from T1 MPRAGE scans used in this study (A, B). This could account for some of the differences (e.g. in the posterior cingulate) between the mean MS found in these networks.

1. Reliability of control vs SUD group differences
   1. Internal replication

In order to assess the reliability of regional MS differences between the control and SUD groups, the Cambridge dataset was split into four different subsets and control vs SUD t-statistics were calculated for each region of interest again. Resulting t-statistics for each of the 360 regions were correlated between each of the four subsets (Supplementary Figure S2). High consistency was observed between these samples as all correlations except for studies codenamed “Study D” vs “Study B” (r=0.05, p=0.34, p_perm_=0.24) were significantly greater than zero (p<0.05). The association between the t-statistic in the “Study D” and “Study C” samples was also low, although it did reach statistical significance (r=0.11, p=0.032, p_perm_=0.038). “Study D” sample was the smallest of the four data subsets (n=65), potentially resulting in lower power to detect SUD-related MS differences. All other correlations were significant at p_perm_<0.001.


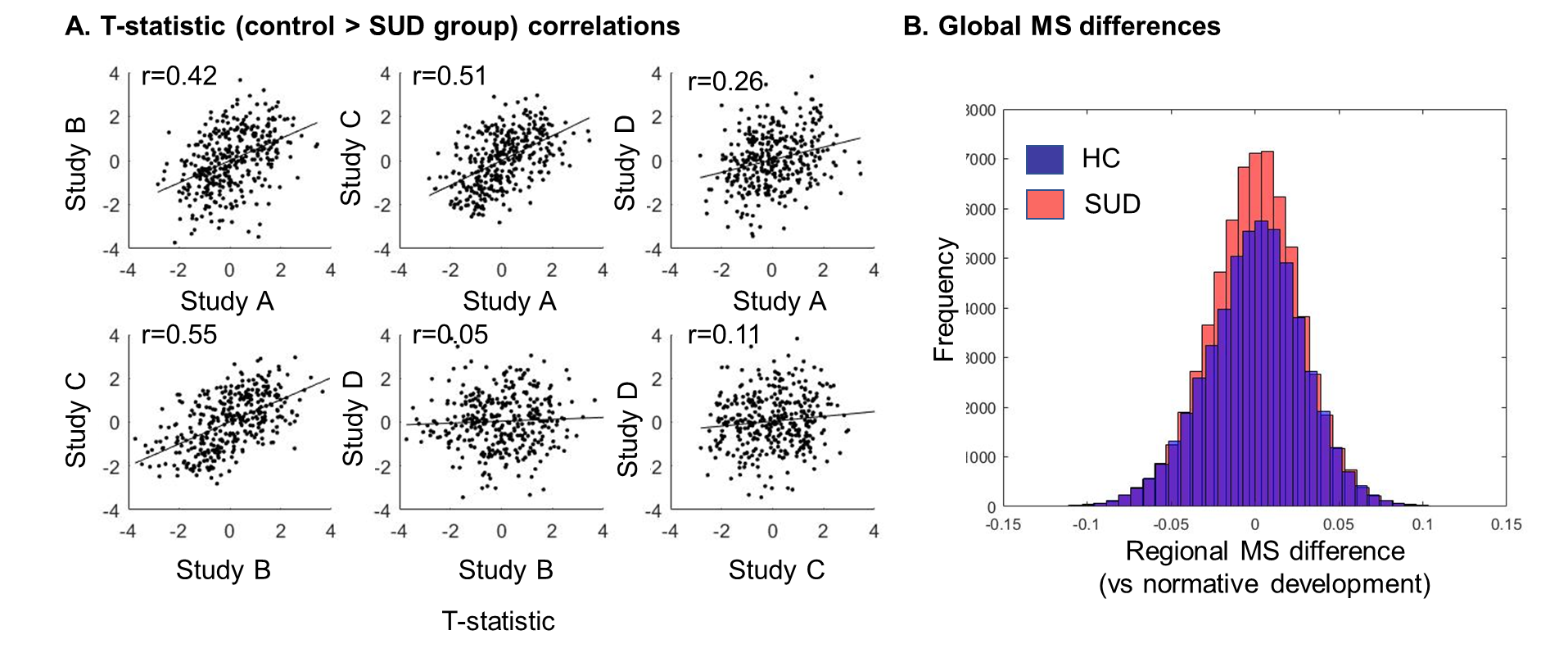


Supplementary Figure S2: A high degree of internal consistency was found across datasets (A). Control vs SUD t-statistics for each of the 360 regions of interest correlated highly between different studies codenamed “Study A”, “Study B”, “Study C” and “Study D”. Study D showed the worst consistency with the other datasets. (B) Regional MS deviations from normative development show a normal distribution of residuals in both control and SUD groups, validating the parametric t-testing approach.

- 1. External replication with Rockland sample

In order to test the robustness of the results found in the Cambridge samples and to ensure that the external Rockland dataset was a suitable normative sample, several comparisons between the Cambridge control and SUD groups and the Rockland sample were ran.

Firstly, global MS differences were compared across the three groups, showing no significant differences in global MS (Supplementary Table 3). Secondly, a subset of the Rockland sample aged 20-60 years old was selected to match the age range in the SUD group. T-statistics were calculated between the Rockland (20-60 years old) group and the control group and between the Rockland (20-60 years old) group and the SUD group. While only 28 regions showed significant MS differences between control and Rockland participants, there were 97 regions that showed significant MS differences between stimulant users and the Rockland sample (p_FDR_<0.001). Further, t-statistics comparing the control and SUD groups were strongly correlated with the t-statistics comparing the Rockland (20-60 years old) and SUD groups (Supplementary Figure S3).


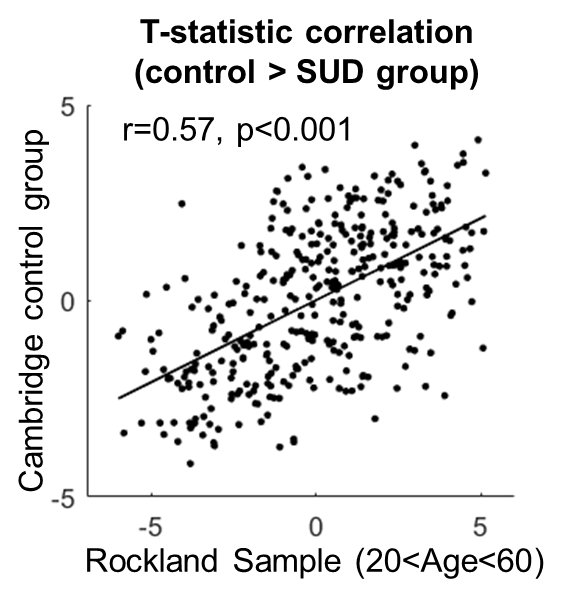
Supplementary Figure S3. Rockland sample vs SUD t-statistics are strongly correlated with the control vs SUD t-statistics (p_perm_ = 3.3*10^-4^), suggesting that the publicly available Rockland dataset provides a consistent control group. Supplementary table 4 also provides a list of significantly different ROIs.

- 1. Permutation testing

Reliability of the MS differences between the Cambridge control and SUD groups was confirmed using permutation testing. More specifically, by permuting group labels and recalculating the difference between the two groups for 5,000 times, we found 60 regions of interest with a significant group differences in MS (Figure S9). These regions included 43 regions identified as significantly different between the control and SUD groups using FDR corrected (p<0.01) t-tests (Figure 1). The overlap in these regions of interest illustrates the robustness of MS differences.

Reliability of the correlation between mean MS in control group and the t-statistic assessing the group difference was tested by correlating t-statistics obtained from permuted group labels in 5000 iterations with the mean control MS. Permuted p-value indicated statistical significance of the correlation (permutation p=0.005). We also used the spin test ^12^ (https://github.com/spin-test/spin-test), but found that regular permutation testing resulted in more stringent p-values and thus report regular permutation p-values.

1. Cross-sectional Trajectories and MS deviation consistency with ageing
   1. Overview of several approaches to testing deviation from normative ageing


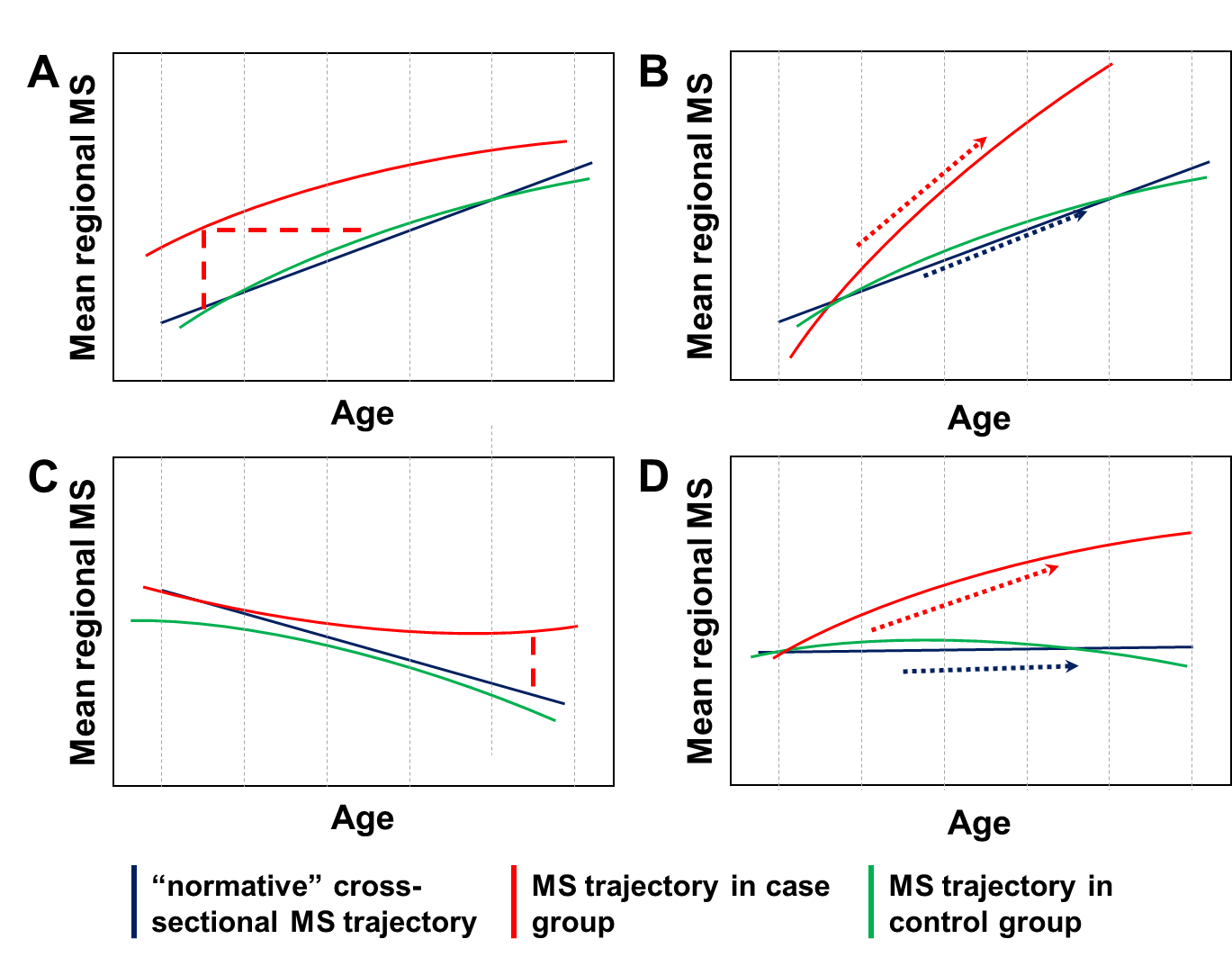


Supplementary Figure S4. Two approaches to assessing accelerated ageing. Panels (A) and (C) showcase the approach we took of comparing deviations from normative trajectories (shown in dark blue). Panels (B) and (D) show an alternative approach, which tests for interactions between age and group. The trajectories are conceptual in nature.

Multiple approaches are available to testing different trajectories in case and control groups. As shown in Figure S4, we constructed normative trajectories in the Rockland sample (blue line) and then tested for significant deviations in SUD cases and in healthy controls (shown in red and green, respectively). We split the sample into 5 age groups (separated by vertical grey lines). In each of these subgroups we tested for significant deviations with a stringent multiple comparison correction. Whenever we found significant deviations, we checked whether those deviations were going in the same direction as the slope of the normative trajectory (MS ~ age). As shown in Figure S4A, this would mean that on average, regional MS of younger cases resembles the regional MS of older controls. This intuition underlies our interpretation of the significant deviations as consistent with accelerated ageing. Note that our approach does not take into account the age-related MS slopes. It also considers each age “bin” groups separately, whereby only one of the age group may show significant deviations (e.g. in Figure S4C).

An alternative way of testing for abnormal ageing effects focuses on the slopes of the MS trajectories in healthy controls and in case groups. A significant group x age interaction on MSN could be indicative of accelerated ageing if the trajectory in the case group resembles an exaggerated version of the trajectory in controls (e.g. Figure S4B). However, in many cases a significant interaction will not be informative of abnormal ageing (e.g. Figure S4D), especially if the relationship between MS and age does not have a pronounced direction in the normative group. This latter approach may also be more restrictive since it’s testing for differences in the relationship between age and MS between cases and healthy controls. Our approach on the other hand is more sensitive, since it considers individual age groups separately.

Further, predicted vs actual brain age gap is an established approach to operationalizing brain ageing ^13^. We present the results of the predicted vs actual brain age gap analysis in Figure S9 and propose that our main analysis (shown in Figure 3) can help localize the effects related to abnormal brain ageing to specific brain regions.

- 1. Construction Method & Consistency Definition

In order to test whether MS changes in the SUD group were consistent with an abnormal ageing profile, we constructed healthy MS cross-sectional trajectories using data from healthy individuals in the Rockland sample (Supplementary Figure S5). Second order polynomial models were selected as they provided best fit to the data (Supplementary Materials Section 2.3). Next, MS differences were calculated for each participant *p* in the Cambridge SUD and control groups as follows:

$${MS difference(p)}_{region}= {observed MS(p)}_{region}-{predicted MS(p)}_{region}$$

${predicted MS(p)}_{region}$=$a_{region}{*(age\left( p \right))}^{2}+b_{region}*(age\left( p \right))+c_{region}$

where *a_region_*, *b_region_* and *c_region_* were fitted for each region based on the Rockland participants’ cross-sectional trajectories (*polyfit* and *fit* functions, MATLAB R2016b). Individual MS differences from control and SUD participants were grouped into five different age categories and one-sample t-tests with FDR correction (p_FDR_<0.01) were used to threshold for statistical significance.

Having identified regions with significant deviations from healthy brain development in both Cambridge groups, we then aimed to determine whether these deviations are consistent with abnormal ageing. To achieve this goal, we first classified the cross-sectional trajectories into four different categories: increasing, decreasing, convex and concave. The first derivative of the polynomial model (approximating the MS trajectory) was used at age 27 and 60 to assess the gradient (upward or downward trend) of the polynomial in early and late life and group the regional trajectories accordingly:

$$f\left( x \right)=ax^{2}+bx+c$$

$$f'\left( x \right)=2ax+b$$

This allowed for an easily interpretable classification of the trajectories. While other classification methods such as hierarchical clustering on the free parameters of the polynomial (*a*,*b*,*c*) were explored, they did not yield the desired interpretability of the above method.

Given the trajectory classification, significant positive MS deviations from healthy trajectories in a given age group were deemed consistent with abnormal ageing if the underlying trajectory was increasing in that age group and inconsistent otherwise.

Group boundaries were selected to ensure sufficient power in each of the groups. Many mid-life participants were represented in our sample, allowing us to split those aged 30-45 into three separate age groups. However, fewer participants aged 20-30 or 45-60 were found in our sample, thus leading us to form groups of ages 20-30 ad 45-60, respectively. We chose to split participants in different age groups rather than look at age as a continuous variable for two reasons. Firstly, assessing cross-sectional trajectories in a brain-wide association study requires larger datasets such as the Rockland sample in order to identify reliable brain-behavior associations. Secondly, we wanted to leverage the significant deviations from normative cross-sectional MS trajectories in each of the age groups separately, thus forming separate age groups.

In order to assess that a trajectory was increasing in an age group, we checked that either it was a) increasing across lifespan or b) that it was convex and had its turning point at an age that was greater than the maximum age in the group under consideration or c) that it is was concave and had its turning point at an age that was lower than the minimum age in the group under consideration. Conversely, significant negative MS deviations were deemed consistent if the underlying trajectory was decreasing in that age group and inconsistent otherwise. In order to assess that a trajectory was decreasing in an age group, we checked that either it was a) decreasing across lifespan or b) that it was concave and had its turning point at an age that was greater than the maximum age in the group under consideration or c) that it is was convex and had its turning point at an age that was lower than the minimum age in the group under consideration.

To make sure that the deviation assessment was reliable, we plotted the MS trajectories and age category-specific deviations in control and SUD groups in Supplementary Figure S8. Most deviations were either increasing or decreasing, facilitating the interpretation of the MS deviations. In those regions that showed decreasing MS with age, having a mean MS lower than what would be expected given the age category indicates abnormal ageing. Conversely, in regions showing increasing MS with age, having an increased MS compared to what would be expected given the age category also indicated abnormal ageing. Further, since the consistency definition was driven by late-life changes in MS, it did not create any issues for the significant deviations from convex or concave trajectories in the 40-45 age group.


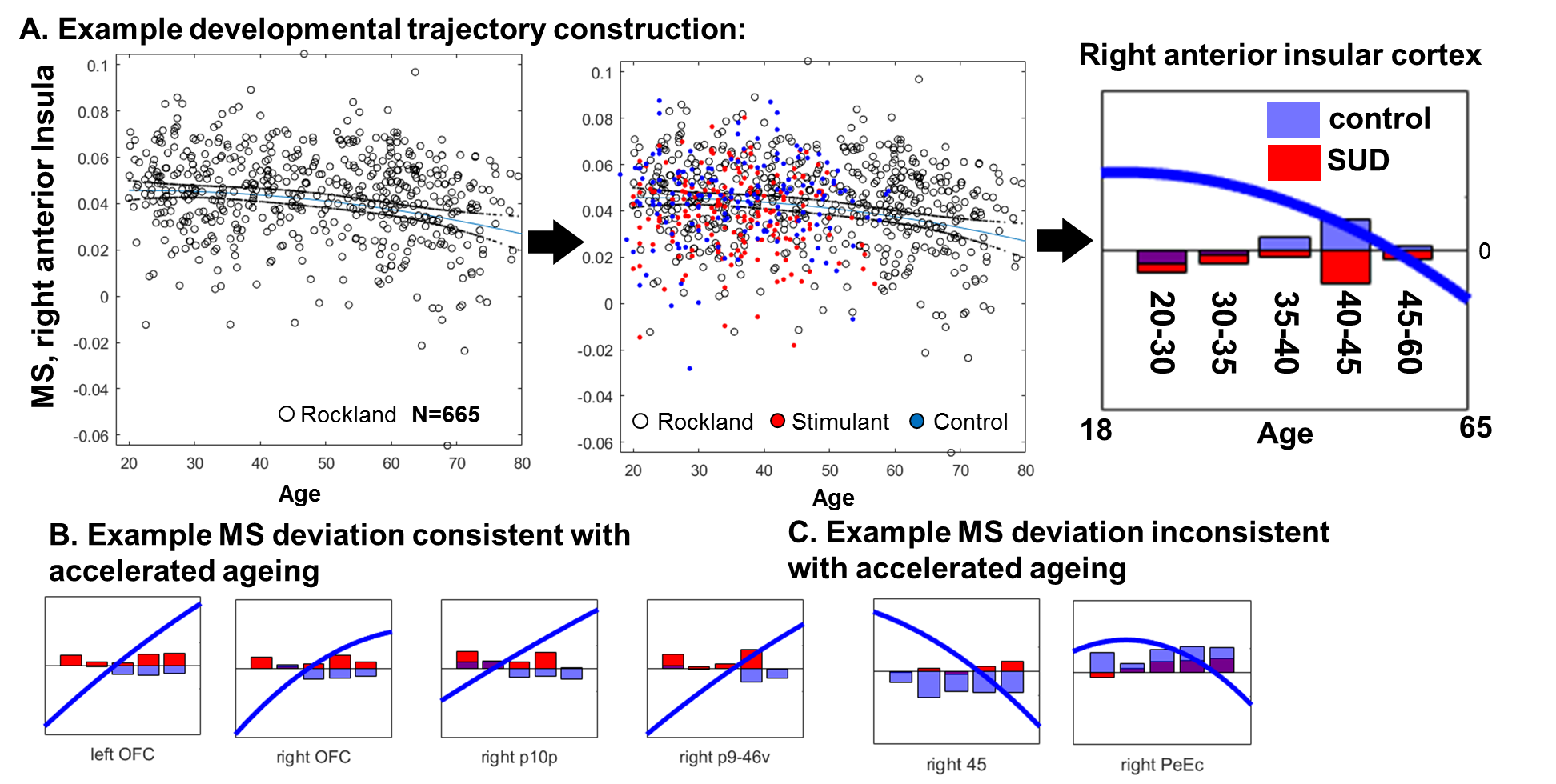


Supplementary Figure S5: Example of the method used to construct cross-sectional trajectories (A) and to define what constitutes a deviation consistent (B) or inconsistent (C) with abnormal brain ageing. In the top right figures the blue line represents the cross-sectional developmental trajectory for the anterior insular cortex, while the red and blue bars represent the age category-specific mean MS deviations from the cross-sectional trajectory in SUD and control groups, respectively.

- 1. Model fit

Model fit was compared using adjusted r^2^ values. While r^2^ measures the proportion of variance in the outcome variable (regional MS) explained by the predictor (Age), adjusted r^2^ adjusts this value to account for model complexity and thus can drop below zero. Whereas the regular r^2^ fit from a model is calculated using the proportion of the sum of squared errors (SSE) out of the sum of squared total (SST), adjusted r^2^ also includes a term that penalizes for the number of model coefficients. The equations for r^2^ are shown below:

r^2^ = $\frac{SSR}{SST}$ = 1 − $\frac{SSE}{SST}$

r^2^_adj_ = 1 − $\frac{(n-1)}{(n-p)}*\frac{SSE}{SST}$

Whereby n is the number of observations, and p is the number of regression coefficients (<https://uk.mathworks.com/help/stats/coefficient-of-determination-r-squared.html>) and SSR is the sum of squares regression. When SSE/SST is close to one, i.e. when almost all of the variance is attributed to error, and the model includes an intercept and e.g. a linear and a quadratic term (p=3), $\frac{(n-1)}{(n-p)}$ becomes greater than 1. This can lead to adjusted r^2^ values that are below 0.

Both quadratic and cubic (2^nd^ and 3^rd^ order polynomials) significantly improved model fit compared to the linear model (1^st^ order polynomial). Since the added complexity of the 3^rd^ order polynomial did not improve model fit, 2^nd^ order models were used for all regions (Supplementary Figure S6).


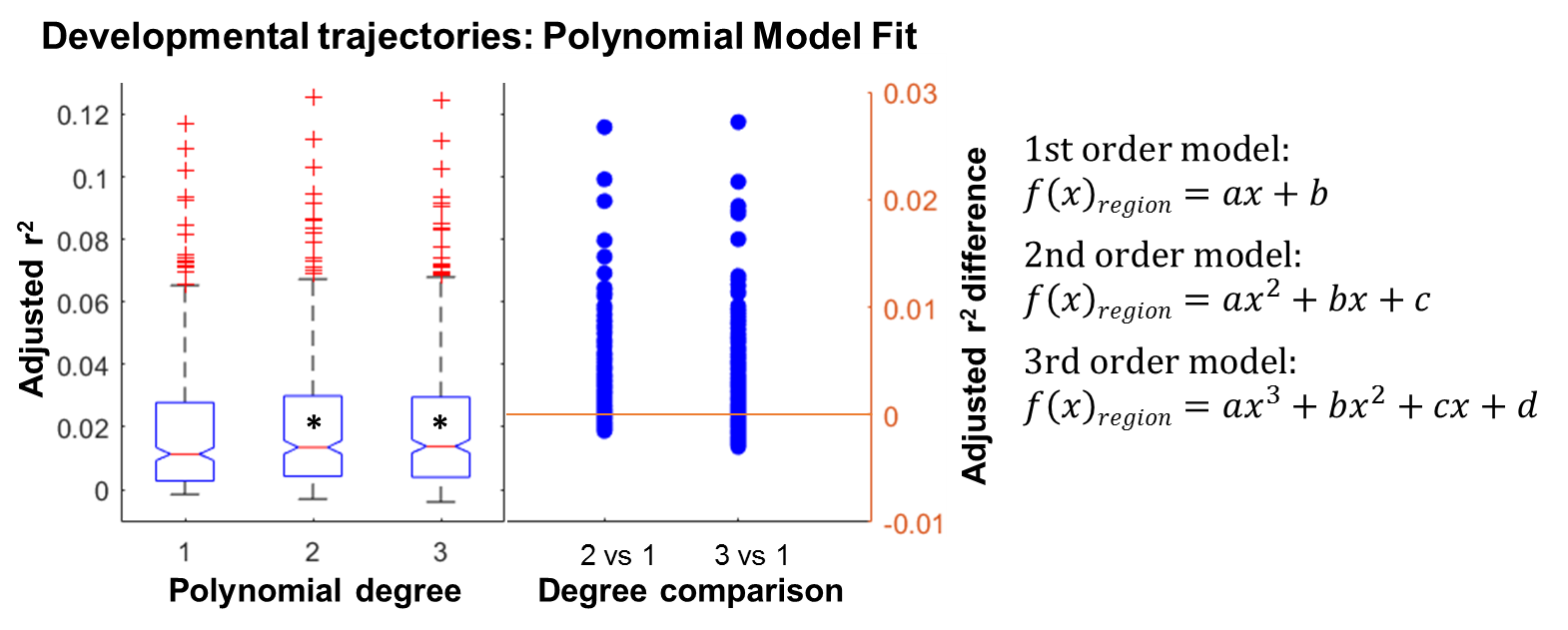


Supplementary Figure S6. Polynomial model fit and comparisons. Left panel shows the distributions of adjusted r^2^ for each polynomial degree, while the right panel shows the distribution of the difference scores (on the right y axis). 2^nd^ order polynomial models were used to model cross-sectional life span MS development. 48 regions showed adjusted r^2^ values below 0 and were not included in the subsequent MS deviation analysis. *p<0.001, paired samples t-test, each datapoint represents one of the 360 regions of interest. For 2^nd^ vs 1^st^ order polynomial comparison, t_359_=5.5; for 3^rd^ vs 1^st^ order polynomial comparison, t_359_=4.8, p<0.001; for 3^nd^ vs 2^nd^ order polynomial comparison, t_359_=0.12, p=0.8.

1. MS deviation from normative cross-sectional trajectories in control and SUD groups

In contrast to the large number of significant MS deviations in the SUD group, only very few significant MS deviations were found in the Cambridge control group (Supplementary Figure S7).


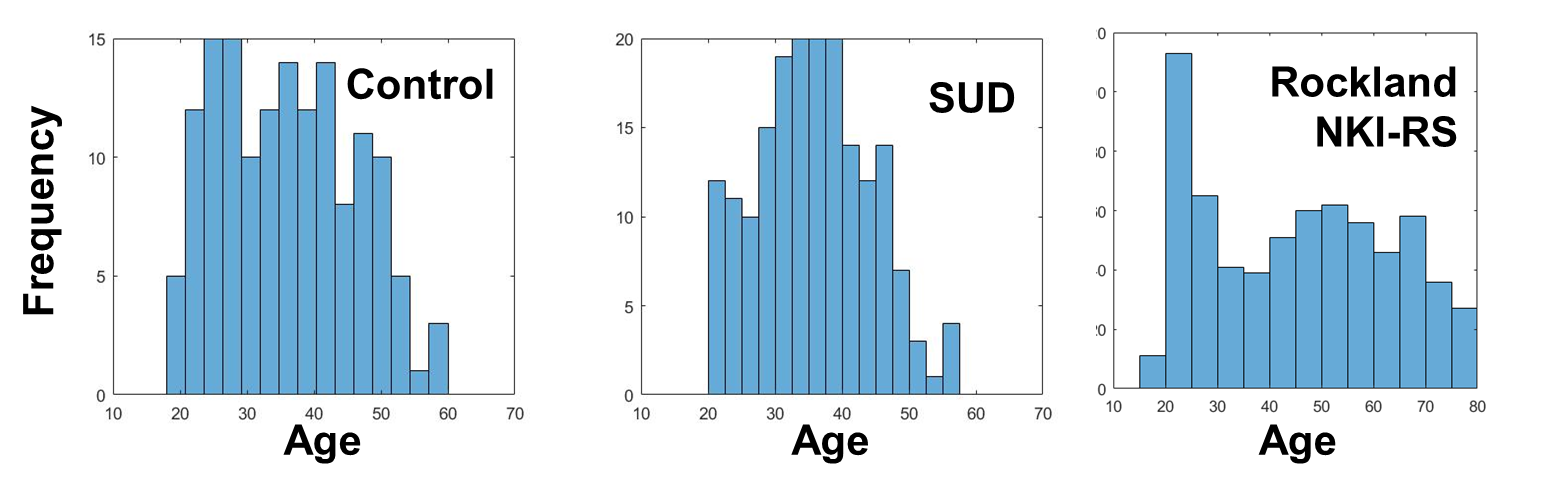
Supplementary Figure S7a. Age distributions of each dataset used in the above analyses (controls, SUD, Rockland).


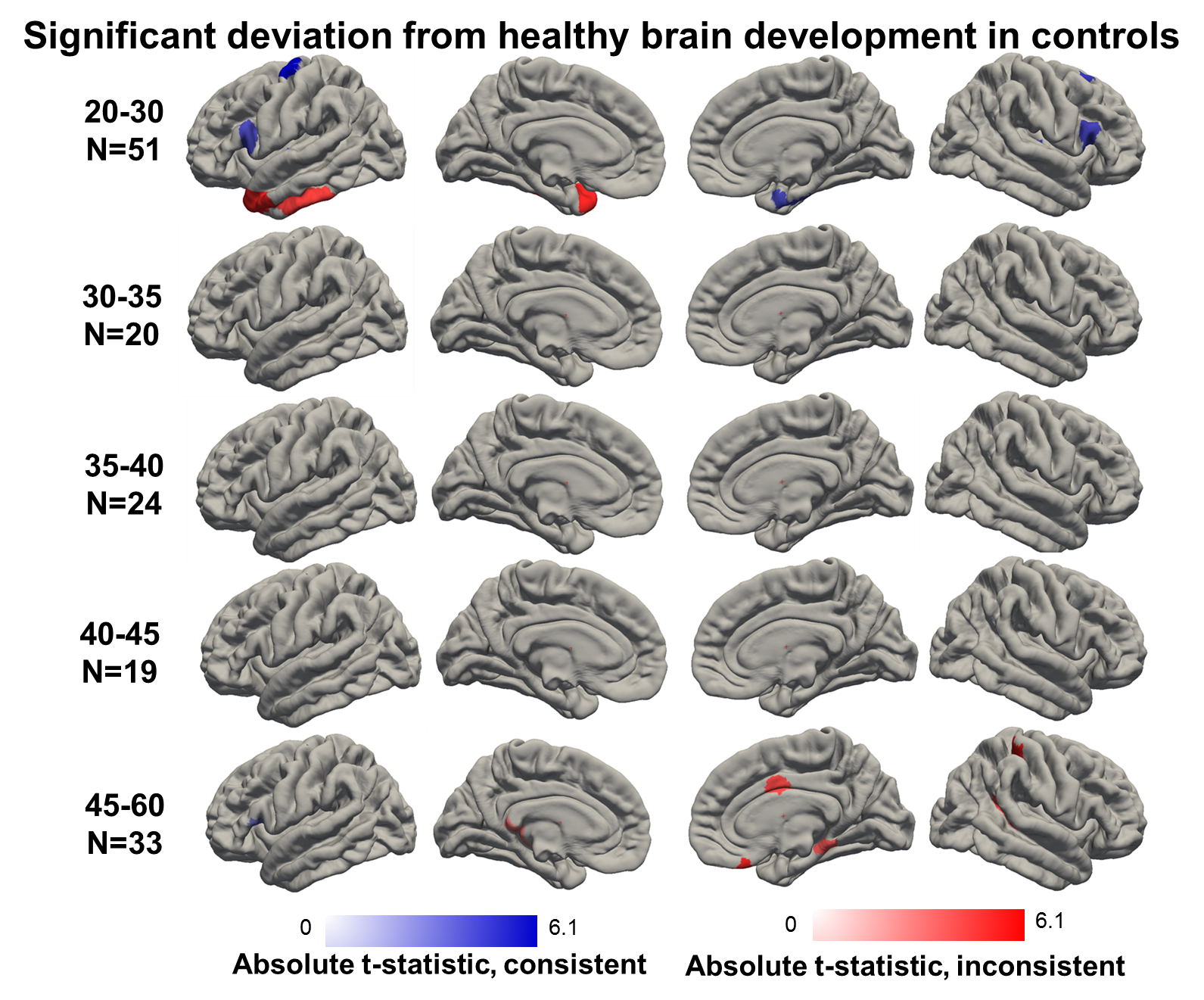


Supplementary Figure S7b. Significant deviations from healthy brain development (in the Rockland sample) in controls. Significance was determined using one-sample t-tests, FDR corrected at p<0.01. Five age categories comprised 20-30, 30-35, 35-40, 40-45 and 45-60 year olds. Regions whose trajectories provided a poor fit to the data (adjusted r^2^<0, Supplementary Figure S6) were excluded. As expected, compared to the MS deviations in the SUD group (Figure 3), controls show only marginal deviations from healthy brain development.


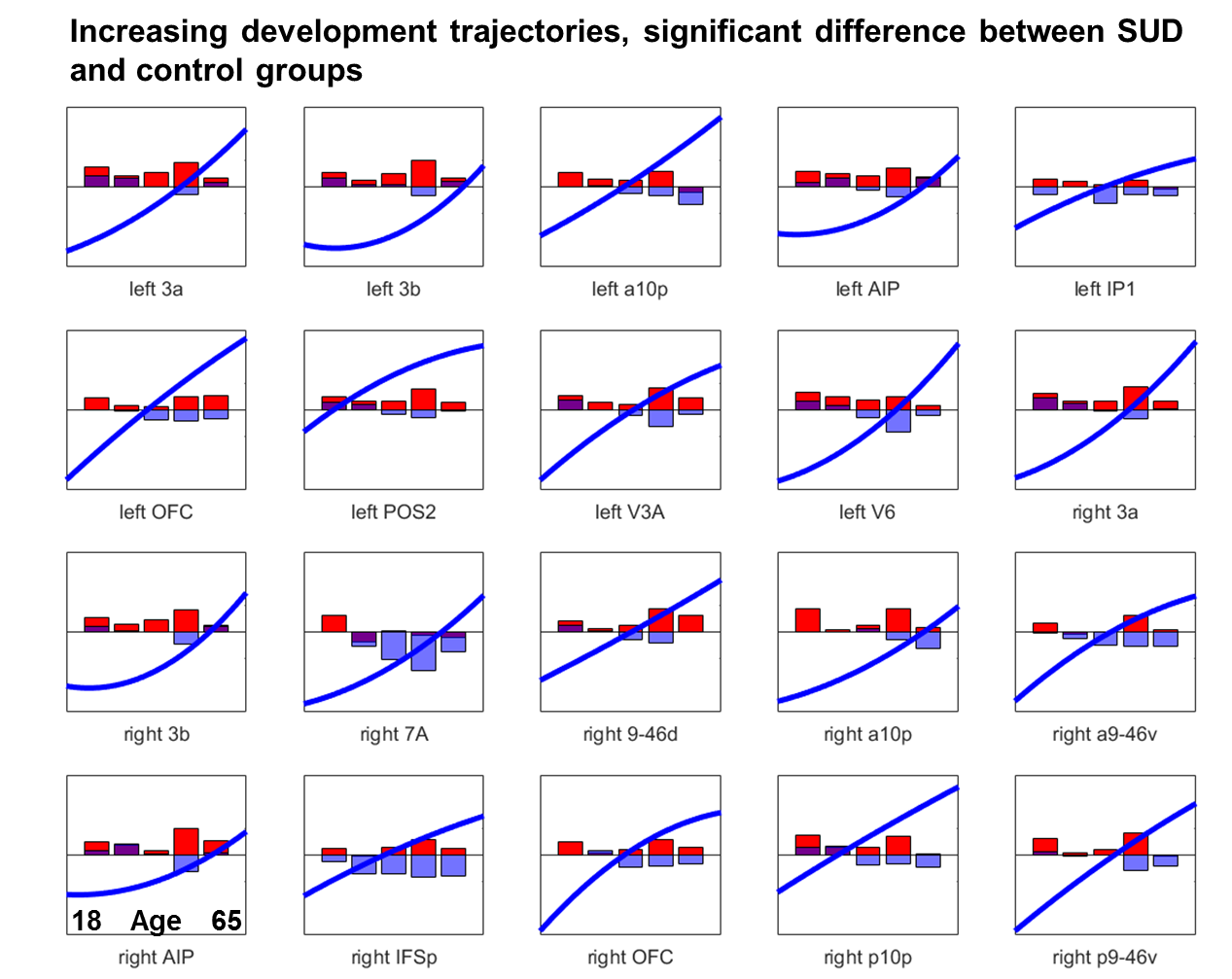
 Supplementary Figure S8.1: Increasing cross-sectional trajectories (blue lines) as well as MS deviations in control (blue bars) and SUD groups (red bars) for each age group (20-30, 30-35, 35-40, 40-45, 45-60). Only those regions are shown that survived multiple comparison correction in the control vs SUD comparisons (p_FDR_<0.01). Importantly, SUD group shows deviations consistent with an abnormal ageing profile.


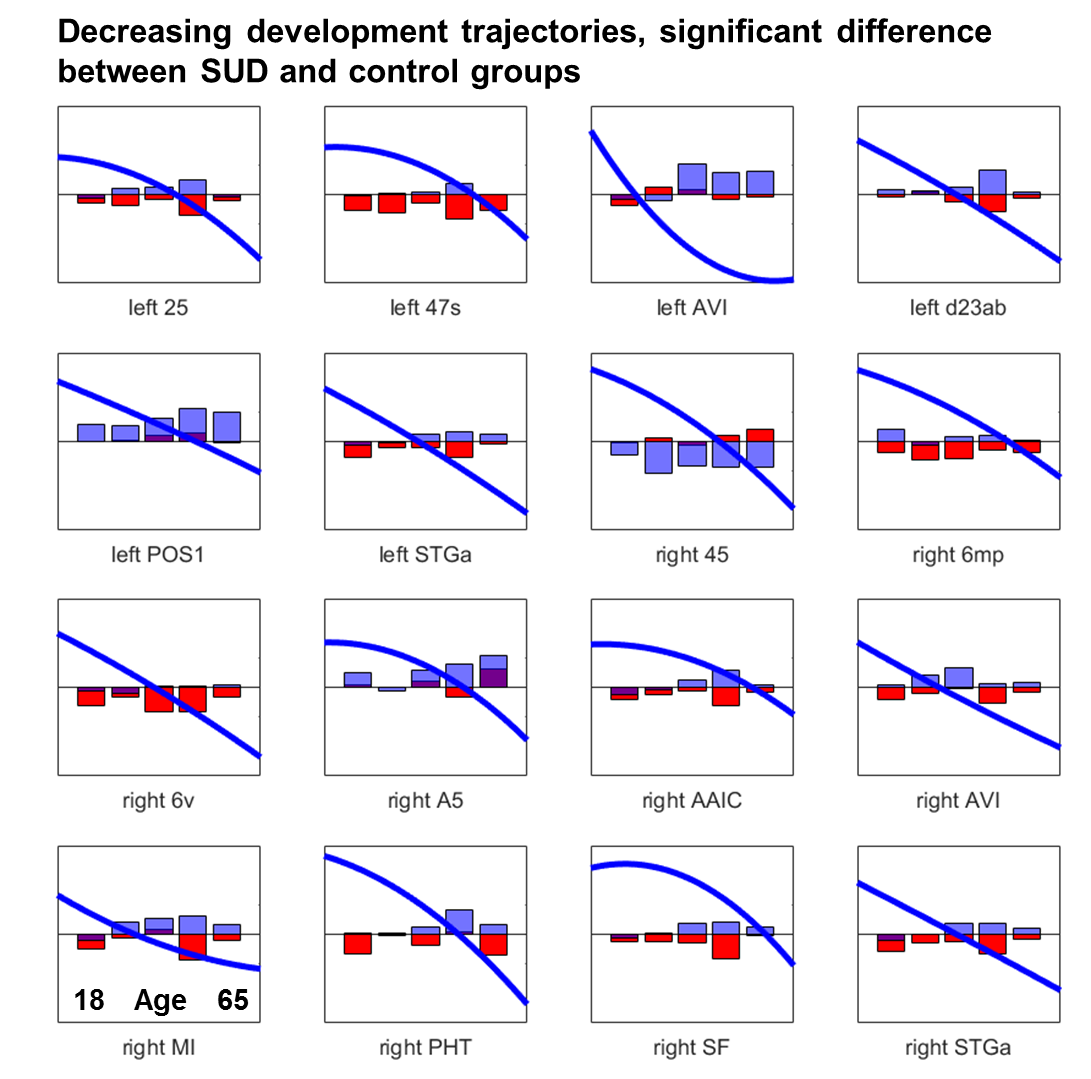


Supplementary Figure S8.2: Decreasing cross-sectional trajectories (blue lines) as well as MS deviations in control (blue bars) and SUD participants (red bars) for each age group (20-30, 30-35, 35-40, 40-45, 45-60). Only those regions are shown that survived multiple comparison correction in the control vs SUD comparisons (p_FDR_<0.01). Importantly, SUD participants show deviations consistent with an abnormal ageing profile.


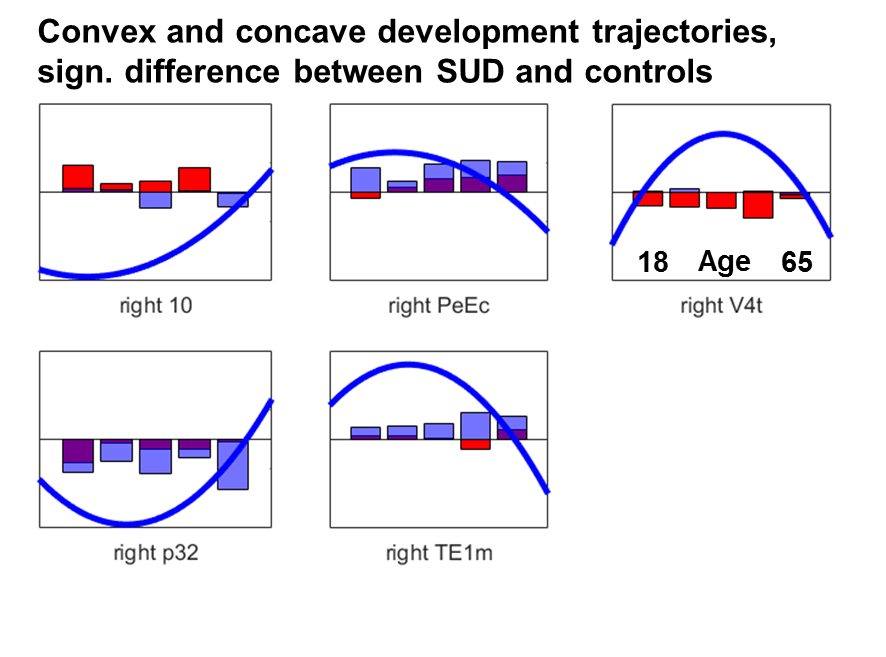
 Supplementary Figure S8.3: Convex and concave developmental trajectories (blue lines) as well as MS deviations in control (blue bars) and SUD participants (red bars) for each age group (20-30, 30-35, 35-40, 40-45, 45-60). Only those ROIs are shown that survived multiple comparison correction in the control vs stimulant comparisons (p_FDR_<0.01). Importantly, stimulant users show deviations consistent with an abnormal ageing profile.

1. Regional Specificity of significant control vs SUD group differences

In order to assess the specificity of the impairments seen in the SUD group, we compared previous MS t-statistic maps ^10^ with our case-control findings (Figure S9). While we found some overlap in the right superior temporal lobes and the premotor cortex, stimulant users (unlike the schizophrenia group) show significant differences in the OFC/IFG and in the mPFC areas that are not seen in the schizophrenia group. As expected, both studies find that the most interconnected regions are also those with the most significant differences between control and clinical groups. This finding is consistent with previous reports of the most interconnected regions in the human connectome being also the most likely to be affected in different brain disorders ^14^. The overall pattern of deviations is thus similar between our SUD findings, schizophrenia ^10^ and major depression ^11^, pointing to a shared neurobiological mechanism of these psychiatric disorders ^15^.


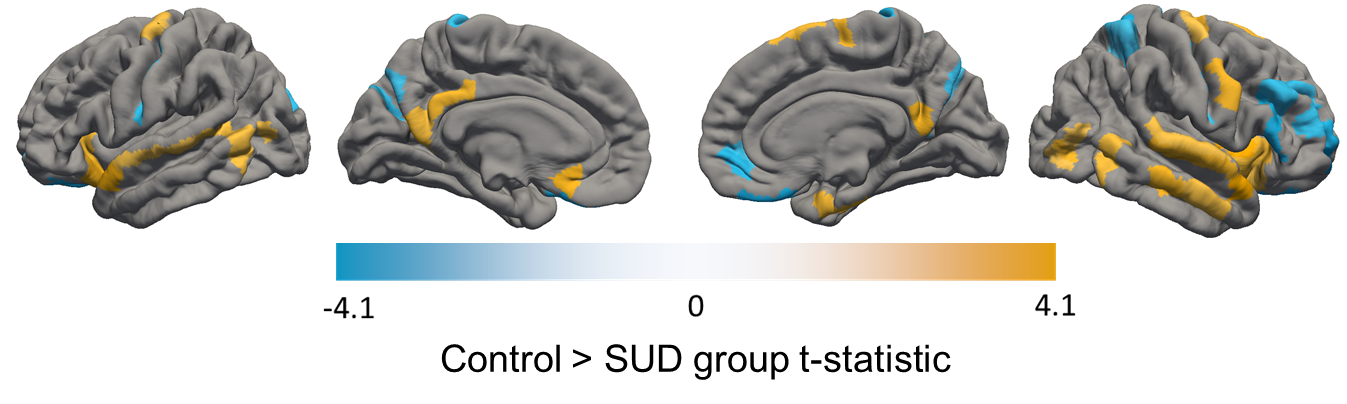


Supplementary Figure S9. Significant case-control differences in SUD (bottom panel). We show 60 regions significant at P_PERM_<0.01 using permuted independent samples t-tests.

1. Stimulant dependent individuals show greater brain age gaps than healthy volunteers

We next used the Rockland sample to build a lasso-regularized regression model predicting a given participant’s age from their morphometric similarity matrix. The resulting lasso regression model used 10-fold cross-validation for optimizing regression parameters and was able to explain 62.4% of variance in age of the Rockland sample participants (Supplementary Figure S10A) by keeping 124 regional morphometric similarity variables as predictors and setting the remaining 236 regression weights to 0. We used permutation testing (n=1000 lasso regressions on permuted data) to test the significance of this model, assessing the amount of variance in age explained by the regression model.

We then applied this model to estimate the predicted age of the Cambridge healthy volunteers and the stimulant dependent participants, finding a significantly greater estimated biological age in the SUD than HC (t_329_=2.47, p=0.014, Supplementary Figure S10B, S10C). When splitting the sample into several age groups, we observed the biggest group effects on differences between the observed and predicted biological age in the youngest group (20-30 years of age, t_93_=-2.38, p=0.02; mean group difference=7.4 years, 95%CI [0.8 9.3]) and in the older group (40-45 years of age, t_40_=1.61, p=0.11; mean group difference=4.3 years, 95%CI [-1.1 9.6]). SUD showed greater biological ages (measured as the difference between their predicted and observed ages) compared to HCs. No significant differences between HC and SUD were seen in the groups aged 30-35, 35-40 and 45-60 years old (p<0.1).


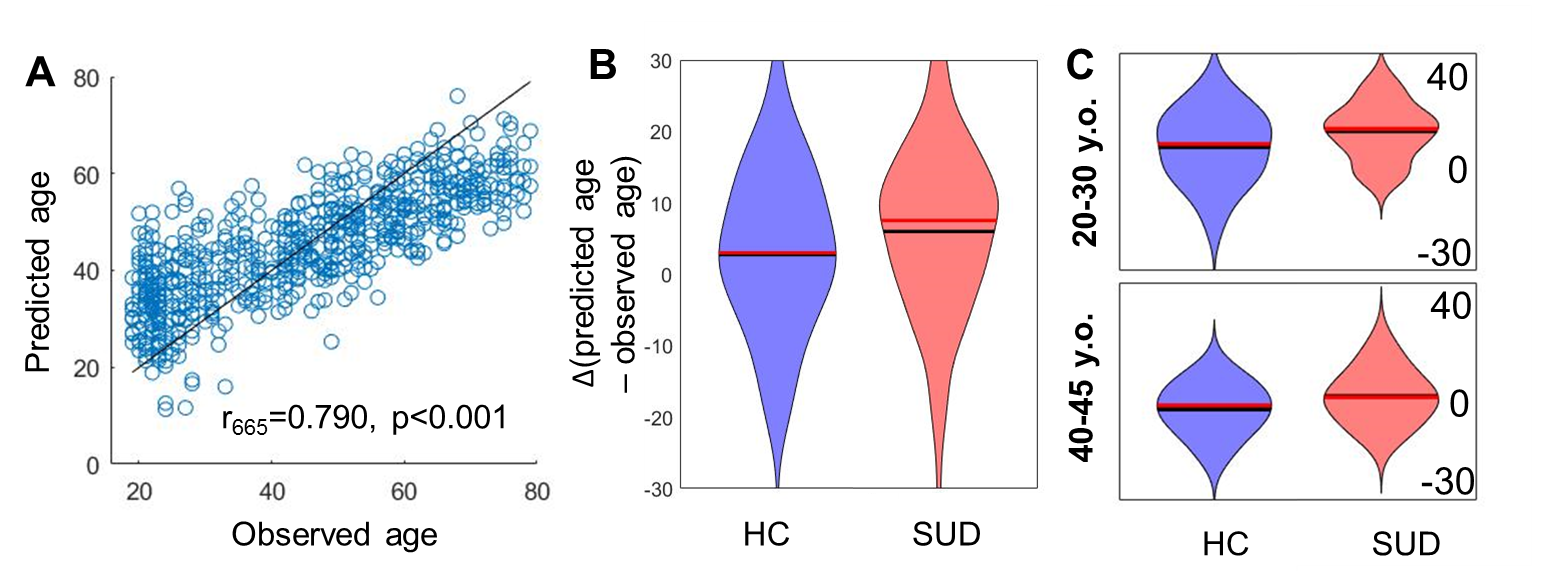


Supplementary Figure S10. Quantification of ageing effects in stimulant dependent participants. (A) A lasso regression model predicted 63.4% of variance in age in the Rockland sample that it was trained on. (B) When the regression model was applied to Cambridge healthy control and stimulant dependent individual groups, HC showed significantly smaller differences in their actual age and the predicted biological “brain age” than SUD (mean deviation in HCs = 2.7 years, mean deviation in SUD=6.0 years; group difference of 3.3 years, 95%CI [0.66, 5.88] years). (C) The biggest differences in the predicted vs observed age were found in the 20-30 year old (p_uncorrected_=0.02) and 40-45 year old (p_uncorrected_=0.11) groups. Means are shown as black lines and medians are shown as red lines.

The results of this regression analysis are consistent with the regional morphometric similarity patterns reported above as they show abnormal brain ageing, especially in the youngest age groups (20-30 years old). Using a separate method, we provide internal replication and extend the conclusions by quantifying biological ageing effects using all the cortical regions of interest. The effects of ageing are likely stronger when analysing specific brain regions in isolation compared to the effects found in this whole-brain analysis as stimulant use affects only a specific subset of brain regions in a way that is consistent with abnormal ageing.

Supplementary References

1. Ersche KD, Jones PS, Williams GB, Turton AJ, Robbins TW, Bullmore ET. Abnormal Brain Structure Implicated in Stimulant Drug Addiction. *Science (80- )*. 2012;335(6068):601-604. doi:10.1126/science.1214463

2. Ersche KD, Hagan CC, Smith DG, Jones PS, Calder AJ, Williams GB. In the face of threat: Neural and endocrine correlates of impaired facial emotion recognition in cocaine dependence. *Transl Psychiatry*. 2015;5(5). doi:10.1038/tp.2015.58

3. Ersche KD, Acosta-Cabronero J, Jones PS, et al. Disrupted iron regulation in the brain and periphery in cocaine addiction. *Transl Psychiatry*. 2017;7(2). doi:10.1038/tp.2016.271

4. Sheehan D, Lecrubier Y, Sheehan K, et al. The Mini-International Neuropsychiatric Interview (M.I.N.I.): The Development and Validation of a Structured Diagnostic Psychiatric Interview for DSM-IV and ICD-10. *22 J Clin Psychiatry*. 1998;59(20):22-33.

5. European Monitoring Centre for Drugs and Drug Addiction. *A Gender Perspective on Drug Use and Responding to Drug Problems*.; 2006. doi:10.5962/bhl.title.42736

6. Nooner KB, Colcombe SJ, Tobe RH, et al. The NKI-Rockland sample: A model for accelerating the pace of discovery science in psychiatry. *Front Neurosci*. 2012;6(OCT):1-11. doi:10.3389/fnins.2012.00152

7. Glasser MF, Coalson TS, Robinson EC, et al. A multi-modal parcellation of human cerebral cortex. *Nature*. 2016;536(7615):171-178. doi:10.1038/nature18933

8. Seidlitz J, Váša F, Shinn M, et al. Morphometric Similarity Networks Detect Microscale Cortical Organization and Predict Inter-Individual Cognitive Variation. *Neuron*. 2018;97(1):231-247.e7. doi:10.1016/j.neuron.2017.11.039

9. Li W, Yang C, Shi F, et al. Construction of individual morphological brain networks with multiple morphometric features. *Front Neuroanat*. 2017;11(April):1-14. doi:10.3389/FNANA.2017.00034

10. Morgan SE, Seidlitz J, Whitaker KJ, et al. Cortical patterning of abnormal morphometric similarity in psychosis is associated with brain expression of schizophrenia-related genes. *Proc Natl Acad Sci*. 2019;116(19):9604-9609. doi:10.1073/pnas.1820754116

11. Li J, Seidlitz J, Suckling J, et al. Cortical structural differences in major depressive disorder correlate with cell type-specific transcriptional signatures. *Nat Commun*. 2021;12(1). doi:10.1038/s41467-021-21943-5

12. Váša F, Seidlitz J, Romero-Garcia R, et al. Adolescent tuning of association cortex in human structural brain networks. *Cereb Cortex*. 2018;28(1):281-294. doi:10.1093/cercor/bhx249

13. Kaufmann T, van der Meer D, Doan NT, et al. Common brain disorders are associated with heritable patterns of apparent aging of the brain. *Nat Neurosci*. 2019;22(10):1617-1623. doi:10.1038/s41593-019-0471-7

14. Crossley NA, Mechelli A, Scott J, et al. The hubs of the human connectome are generally implicated in the anatomy of brain disorders. *Brain*. 2014;137(8):2382-2395. doi:10.1093/brain/awu132

15. Caspi A, Houts RM, Belsky DW, et al. The p factor: One general psychopathology factor in the structure of psychiatric disorders? *Clin Psychol Sci*. 2014;2(2):119-137. doi:10.1177/2167702613497473

16. Morgan SE, Seidlitz J, Whitaker KJ, et al. Cortical patterning of abnormal morphometric similarity in psychosis is associated with brain expression of schizophrenia-related genes. *Proc Natl Acad Sci*. 2019:201820754. doi:10.1073/pnas.1820754116
